# Contactless Acoustic Trapping of Hoverflies for Behavioral Studies

**DOI:** 10.1111/nyas.70281

**Published:** 2026-05-18

**Authors:** Thomas Gaillard, Victor Contreras, Dominique Martinez, Stéphane Viollet

**Affiliations:** ^1^ Aix Marseille Université CNRS, ISM Marseille France; ^2^ Universidad Nacional Autonoma de Mexico Instituto de Ciencias Fisicas Cuernavaca Mexico

**Keywords:** acoustic tethering, behavior, fly, free‐fall, insect, levitation, verticality perception

## Abstract

Studying the sensorimotor response of flying insects is usually done in tethered conditions. The righting reflex, for example, is studied with insects that are released either from a rigid or a magnetic tether. The attached tether or magnet may, however, induce proprioceptive biases and behavioral artifacts through physical contact and additional mass or inertia. Here, we propose a contactless tether apparatus based on acoustic levitation. It relies on the emission of ultrasonic waves on both sides of the device, creating standing pressure waves—visualized using the schlieren technique—that enable insect levitation. We demonstrate its effectiveness on living hoverflies *Episyrphus balteatus*. By quantifying the rate of motion of four natural body markers (head, abdomen, wing, and leg) and the wingbeat response, we show that hoverfly levitation can be stable for a long period of time, with minimal behavioral perturbation under ultrasound stimulation. We further illustrate the application of ultrasound tethering to study the hoverfly righting reflex. Beyond hoverflies, ultrasound tethering was applied successfully to levitate other insect species, such as dead *Drosophila* and ants. More broadly, ultrasound tethering might be relevant in the context of behavioral studies with flying or walking insects.

## Graphical abstract

We present a contactless tethering device based on acoustic levitation. Opposing ultrasonic transducers generate standing waves, creating stable pressure nodes that enable the sustained levitation of hoverflies (*Episyrphus balteatus*). We show that acoustic levitation does not appear to affect hoverfly behavior. We also demonstrate the capability to levitate other insects, including ants and *Drosophila*, highlighting acoustic trapping as a potential noninvasive tool for behavioral and neuroethological studies.

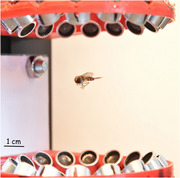

## 1 Introduction

Numerous studies have used tethered flight to investigate the flight behavior of migratory insects [1]. However, tethering severely restricts the degrees of freedom of motion of the insect: the fly can change only its heading by walking on an air‐supported ball or rotate around its yaw axis via magnetic coupling. Moreover, the added mass or inertia due to the coupling can disturb feedback loops involved in visuomotor reflexes and thus modify the flight behavior [1], such as variability introduced in head and wing control by locking the fly in space [2]. We study how free‐falling hoverflies (*Episyrphus balteatus*) use multiple sensory cues like visual, antennal, and proprioceptive cues to generate appropriate commands to the wing and reach a stable steady‐state [3, 4]. Previous studies have shown that the position of light in the visual field is critical to allow the fly to recover from free fall [5]. In addition, antennal cues seem to play a crucial role in the estimation of the vertical direction since we observed, in one of the experiments where antennae were immobilized, that hoverflies flew upside‐down while still actively flapping their wings [4]. The role of leg proprioception prior to take‐off and during flight remains unresolved. To address this, it is desirable to release flies into free fall, either upright or upside‐down, without any prior surface contact, thereby avoiding stimulation of the tarsal reflex [6, 7, 8]. These constraints make free fall a relevant alternative for studying behavior under nonrestrictive conditions.

Acoustic radiation forces (ARFs) represent an alternative for contactless tethering of insects. ARFs arise from momentum transfer from acoustic waves to objects, producing net mechanical effects (push or pull) that depend on the mean‐square fluctuations of pressure and particle velocity in fluid media [9, 10]. In standing waves, ARFs trap particles smaller than half the wavelength (λ/2) at pressure nodes and larger objects at pressure antinodes [11]. Focused ultrasonic standing waves can trap objects at the millimeter‐scale of different materials, making them perfect candidates for studying insects walking or flying in the air without any tether or flight mill [12, 13]. For instance, Xie et al. demonstrated airborne acoustic levitation of live animals smaller than λ/2 (λ = 20.3 mm) using an emitter‐reflector cavity [12]; however, their design relied on short cavity lengths (resonant mode *n* = 3, *L* ∼ *n* λ/2), which technically restricts lateral sample access, which is desirable in many applications.

Recently, the technology of phased arrays of low‐power transducers has been applied to ultrasonic levitation, enabling the design of larger and more versatile cavities [12, 14, 15]. Low‐power transducers driven harmonically can be mounted on opposing spherical caps to generate focused standing waves with lateral confinement. For nonspherical objects levitated in axisymmetric cavities, the ARF can act asymmetrically, producing transverse torques that induce spinning. To avoid spinning, Cox et al. presented a phased‐array spherical cavity to stably trap irregular objects and insects with sizes < λ/2 (λ = 8.6 mm) by time‐multiplexing two acoustic fields (standing wave and twin trap) via electronic phase control [13]. Because standing waves generate large axial forces on small objects at central nodes, alternating in brief periods of time between a standing wave (axial force) and a twin trap (lateral force in one transverse direction), they enable levitation of nonspherical objects without rotational movement.

In a later work, it was demonstrated that the twin trap can be described as a particular transverse pressure‐field pattern [15]. Standing waves of high‐order transverse modes (HOT modes) create more complex patterns than the fundamental (m = 0). HOT modes are distinguished by the m‐nodal planes along the symmetry axis generated by transverse phase dislocations of the emitters (see Ref. [15] for a detailed description). HOT modes enhance levitation across a wider range of shapes and sizes while maintaining rotational stability, including objects larger than λ and with weights in the millinewton range, which characterizes insects like Diptera. As the acoustic field complexity rises, the number of equilibrium positions increases, although they are more difficult to predict due to their dependence on both the incident and scattered field (geometry and size of the object). However, it was demonstrated that HOT modes can stably levitate millimeters‐sized elongated objects (including sizes >λ) without requiring alternating fields [15].

In this study, we demonstrate that acoustic levitation can be used as a contactless tether that does not add mass to the insect's body or require the feedback‐loop control typical of magnetic levitation [16, 17]. As pointed by Rode et al. [18], contact‐free acoustic tethering can be an alternative to direct mechanical confinement and tethering.

We implemented a multi‐emitter levitator based on two opposite arrays of ultrasonic transducers (embedded on concave spherical segments) (Figure 2A) forming an axial cavity [19]. Compared with previous acoustic or diamagnetic devices developed for levitating small animals [12, 20], our levitator is low cost, low power and allows the animal to be filmed from below or above through lateral apertures in the spherical segments (Figure 2A).

**FIGURE 1:**
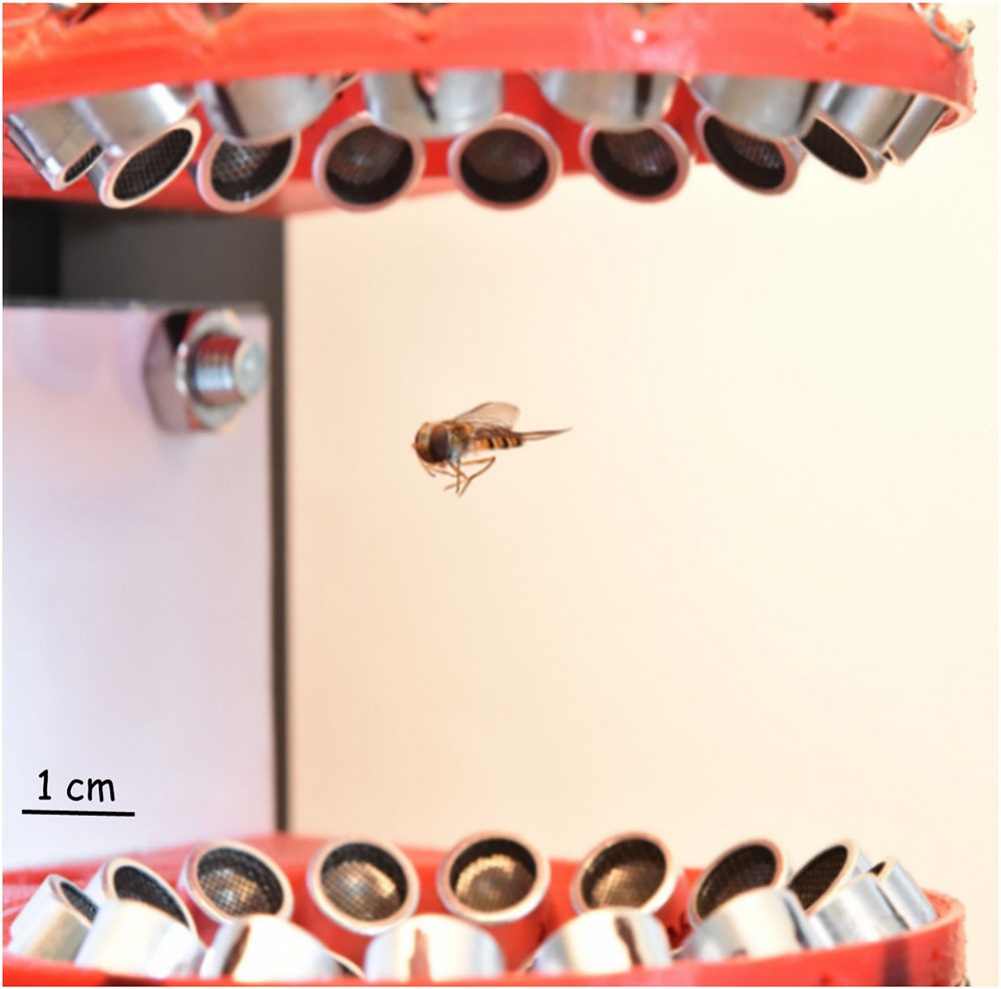
Side view of a hoverfly (*E. balteatus*) levitating in the acoustic levitator.

**FIGURE 2:**
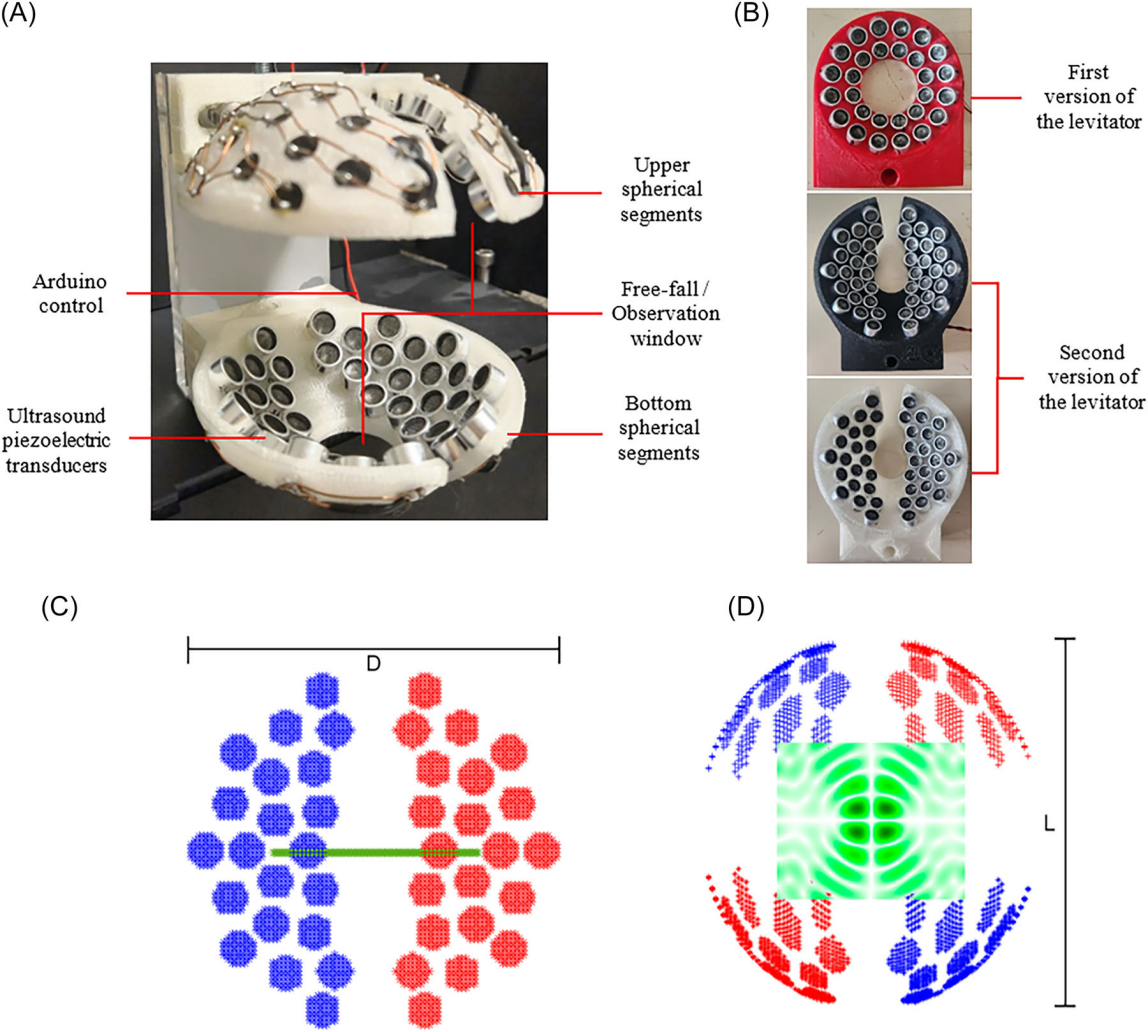
(A) Side view of the ultrasonic levitator consisting of two separated and opposite arrays, each composed of 42 ultrasonic transducers. (B) Acoustic levitator configurations. The initial prototype (red) is shown here for illustrative purposes (see Figure 1). All behavioral experiments were conducted using black and white levitators featuring a lateral aperture. This design modification ensures an unobstructed optical path between the insect and the high‐speed camera, eliminating visual occlusion during image acquisition. (C) Upper view of the geometrical configuration of the transducers placed on one spherical segment. Colors indicate electronic phase signal (blue = 0, red = π‐radians) (D). Side view of the acoustic cavity with an observation plane showing the pressure distribution (green indicates pressure) generated by the arrays.

The ultrasound avoidance reflex is possible in insects endowed with tympanal organs [21]; therefore, a prerequisite to using acoustic levitators for behavioral analysis is that hoverflies are not sensitive to ultrasound or, at least, that their behavior is not disturbed by the presence of ultrasound. As depicted in Ref. [22], there are two types of auditory organs: first, the auditory organs derived from chordotonal organs, which are found on various parts of the insect body; the second type are the pressure‐sensitive tympanal ears, which can operate at a frequency of up to 50 kHz (e.g., 50 kHz in the parasitoid fly *Ormia ochrace* [23]). Unlike some insects such as crickets and praying mantis, which feature the ultrasound avoidance reflex [21], Dlpteran flies hear with tympanal ears and the Johnston's organ, a chordotonal organ in the second segment of the insect antenna [24]. The tympanal ears of flies do not seem to detect the range of ultrasounds that we use (except for the parasitoid fly mentioned above). In addition, their antennal ears seem to be used for communication at short distances and usually operate at frequencies below 1 kHz, but one cannot exclude that the fly's behavior is affected by ultrasound. Therefore, after validating that hoverflies could be held stably in our levitator (Figure 1 and Videos S1–S5), the central question was whether ultrasound can reliably restrain flies at a fixed position prior to release for free fall without affecting their natural behavior. We analyzed finely the movement of the head, tip of the abdomen, tip of a wing, hind leg, and the wingbeat. Once the tethered fly was placed in the acoustic levitator, we analyzed the fly movement with and without the emission of ultrasound. Finally, a complete statistical analysis demonstrated no effect of ultrasound on fly behavior.

## 2 Materials and Methods

### 2.1 Flies

All hoverflies (*Episyrphus balteatus*; De Geer 1776) were obtained from Katz Biotech AG (Baruth, Germany) and were procured at the pupa stage. The adults were subsequently reared in an appropriate flight area and subjected to a 12/12 light cycle. The flight area comprised an entomological breeding cage measuring 72 × 72 × 107 cm. A 30 × 40 cm platform for the emergence of pupae was suspended inside it. The hoverflies were bred in groups of 50 at 25°C and their mean mass was 22.1 mg (SD=5.9 mg). The insects were provided with unlimited access to a mixture of pollen and water, as well as real flowers, to stimulate flight behavior. The flowers were specifically selected for their suitability for rearing hoverflies [25]. *Malva sylvestris* is beneficial for its sugar contribution and its extension of the longevity of hoverflies in captivity. Similarly, *Echium* sp. is advantageous for its lipid contribution. To keep the flies in the middle of the levitator for the control phase (without ultrasound) and prevent injury, a tube of ultrasound‐transparent generic lens cleaning tissue (MC‐5, Thorlabs), with a mass of less than 1 mg, was fixed to the dorsal part of the thorax using an adhesive composed of 50/50 beeswax and rosin. The ability of hoverflies to fly was verified in the rearing cage before and after any manipulation.

### 2.2 Acoustic Levitator

The airborne acoustic levitation was achieved by producing focused standing waves inside a cavity (Figure 2D) made of two arrays of 42 compact ultrasonic sources (piezoelectric transducers MSO‐A1040H07T, Manorshi) (Figure 2A). The 42 ultrasonic sources, operating with a frequency (f) = 40 kHz, and wavelength (λ) = 8.5 mm at standard pressure and temperature (STP), were embedded in 3D‐printed spherical segments (SSs) made of polylactic acid (PLA). The SSs have a radiating surface with a radius of curvature of r=43(±1) mm and a circular aperture with a diameter of D=75(±1) mm. This geometry provides a high numerical aperture, a parameter proportional to the ARF [26]. The ultrasonic sources were arranged in the SSs by dividing them into two identical halves separated by a vertical opening to allow access to the levitating samples. The SSs have horizontal cuts at top and bottom to allow the insects to fall freely.

The levitator was operated to produce focused standing waves having a nodal plane along the axis of symmetry (Figure 2D), which is called twin trap [13] or HOT mode, *m* = 1 [15]. While the fundamental standing wave (*m* = 0) can stably trap small objects usually with spherical symmetry, nonspherical and irregular objects tend to rotate due to the unbalanced forces because of the lack of axial symmetry. The HOT mode, *m* = 1, achieves stable confinement of diverse samples of irregular shapes with sizes >λ and weights above 100 mg, improving the acoustic levitation capacity. To produce HOT mode *m* = 1 in our levitator, the halves were electronically connected to produce waves with a π‐phase difference as indicated by the colors blue and red in Figure 2D. The pressure distribution on a plane inside the cavity that intersects the axis of symmetry is shown in Figure 2D. This pressure distribution was obtained numerically using the method reported in Ref. [27]. The harmonic signal is produced by simple electronics following the protocol outlined in Refs. [14, 28].

Subsequent calibration for resonant conditions was conducted by ensuring precise alignment of the lower and upper SSs and adjusting the distance between them. This is a critical step since resonant conditions can considerably increase the levitation capacity of the device and consequently increase the maximum weight of samples to be levitated [27, 29]. The distance can be adjusted to resonance by monitoring the current consumption of the power supply. A resonant distance corresponds to a local minimum of the electrical current consumption [29]. We used a precise ammeter (Aim TTi model 1908 5.5 digit dual measurement multimeter) to measure a minimum current consumption of $I_{min}∼750$ mA under 15 V (peak signal) at 23°C for a distance of L=87(±1) mm adjusted manually by using the linear stage with standard micrometer (PT1, Thorlabs) attached to the upper SS. Once *L* was determined, the SSs were mounted on an acrylic lateral support to keep *L* fixed for all experiments.

### 2.3 Experimental Setup

**FIGURE 3:**
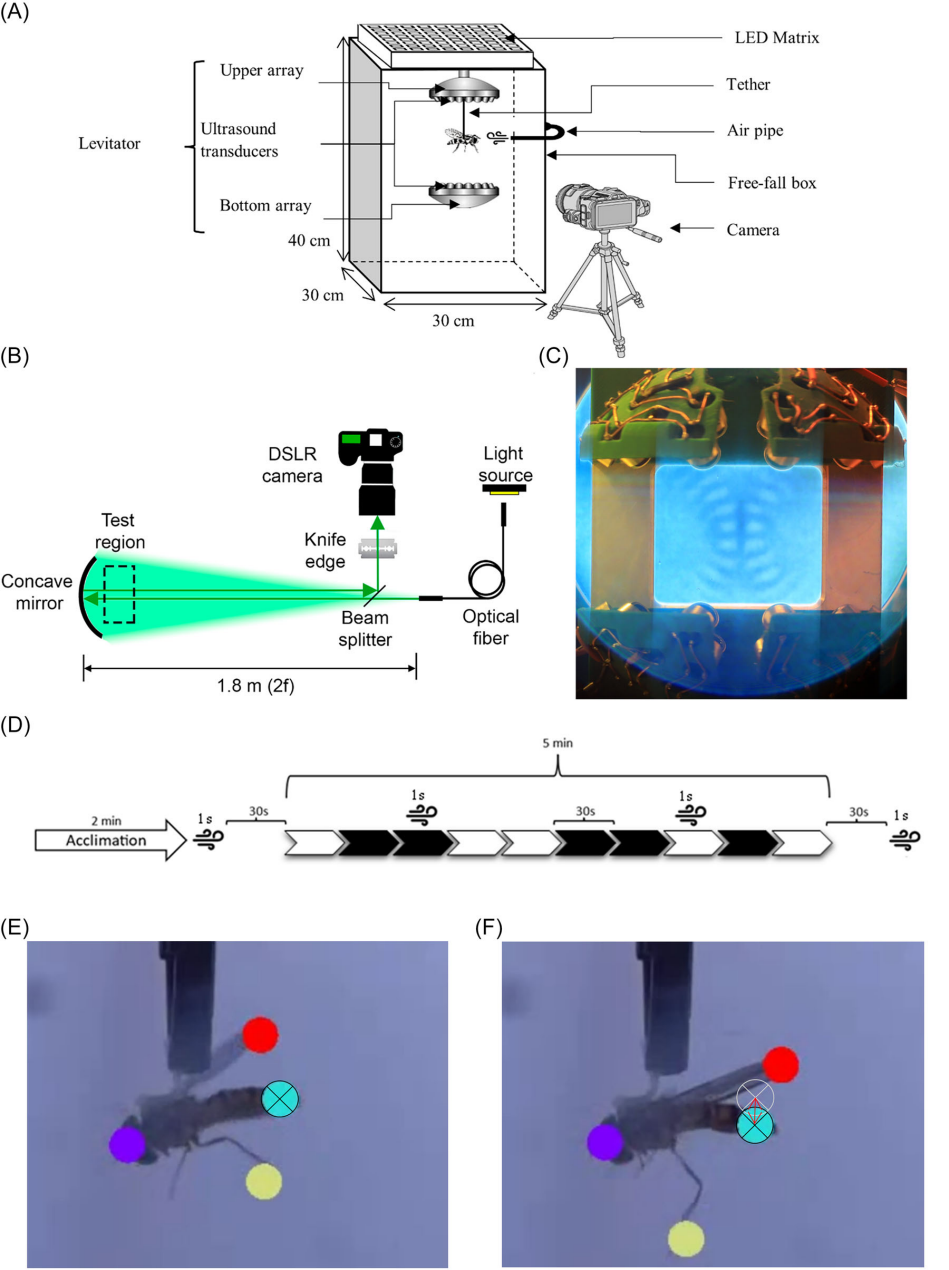
(A) Schematic of the experimental setup used to study the behavior of hoverflies in presence of ultrasound emitted by the acoustic levitator. (B) Schematic of the schlieren device used to display the pressure nodes resulting from the emission of ultrasound by the two domes. Adapted from Ref. [30]. (C) Photographs of the levitator pressure nodes using the schlieren technique. The pressure nodes are represented by the blue level differences. (D) Methodology used to study the behavior of hoverflies subjected to levitator ultrasound. Series of 30‐s when ultrasound was turned on (white slot) or turned off (black slot) and 1‐s air puffs. (E) Photographs of the DLC tracking of hoverfly no. 13 at *t* = 0.55 s (frame 16). The black cross is the current position of the abdomen. (F) Photographs of the DLC tracking of hoverfly no. 13 at *t* = 0.88 s (frame 26). Black cross is the current position of the abdomen, and the light gray cross is the position of the abdomen at frame 16. The double red arrow shows the difference in abdomen motion between 10 frames.

The setup (Figure 3A) is composed of a 40 × 40× 30 cm transparent PVC box illuminated from above by eight LED arrays (Adafruit Rigid 8 × 8 NeoPixel RGBW 19‐Watt), which were positioned to diffuse white light across the top of the box. The side walls and rear wall were uniformly white and diffused. Hoverflies were filmed in the box with a camera (SONY, 60 fps, RIBCAGE RX0) equipped with a Fujinon lens (Fujinon CF16HA‐1 16 mm 1.5MP 1″ f/1.4–f/22 C‐Mount Lens) through a two‐way mirror at full resolution (1920 × 1080 pixels). The mirror allows for observation of the fly without allowing insects to see outside. The acoustic levitator was positioned at the apex of the box, and the hoverflies were restrained in the center of the levitation apparatus by means of a black clamp, which was 3D‐printed with PLA at the laboratory (CAD file available in the Supporting Information). Additionally, we implemented a schlieren setup schematically presented in Figure 3B, because the schlieren effect [30] allows the direct visualization of acoustic pressure distribution and thus enables the direct visualization of trapping positions in the standing waves [31]. The schlieren setup consists of a white light source (MCWHF2, Thorlabs), controlled with a constant current LED Driver (UPLED, Thorlabs) coupled to a fiber optic cable (M14L02, Thorlabs), with a 50 micrometer core used as a point‐like source. A single spherical mirror (D114F900 mm, Amazon) with a focal length of 0.9 m was placed at a distance of 1.8 m (corresponding to twice its focal length) in order to produce a back‐propagated image with the same size as the source. To obtain access to the image plane, a 50–50 beam splitter was used. A knife edge was placed at the image plane to partially block the light and produce the schlieren effect, which was captured with a digital single‐lens reflex (DSLR) camera. Figure 3C shows a schlieren image of the standing wave inside our levitator, having a focused standing wave split in two halves by a vertical nodal plane. According to numerical simulations (Figure 2C), the brighter regions in Figure 3C correspond to the pressure antinodes.

### 2.4 Experimental Method and Data Acquisition

To determine whether the ultrasound disturbed the behavior of the hoverflies, behavioral tests were conducted on 28 individual hoverflies (14 males/14 females). Once the flies had been positioned and maintained in the center of the levitator by the paper tube glued to their thorax (the location of the nodal planes enabling strong levitation), the experiment began with a 2‐min and 30‐s acclimation period without ultrasound (Figure 3D). Subsequently, the 5‐min test phase was started. The latter was divided into five periods of 30 s with ultrasound and five periods of 30 s without ultrasound as a control condition. The order in which the ultrasound was activated or deactivated was determined randomly beforehand. Additionally, air puffs with an average speed of 1.67 m/s were produced by means of a compressor (Dürr Technik SAS‐038 Silent Air System) connected to a pneumatic distributor (CMS‐200, C.I.F Athelec, France). The air puffs lasted approximately 1 s and were directed frontally toward the hoverflies by means of a 3D‐printed white PLA tube (diameter of 5 mm) to trigger the wingbeat reflex [32, 33] at four instants: at *t* = 2 min of acclimation (30 s before the test); during the test without ultrasound; during the test with ultrasound; and finally 30 s after the end of the test. To study the response of the fly to the presence of ultrasound, we analyzed the rate of motion of four natural body markers (Figure 3E; Videos S15 and S16): the legs, abdomen, wing tip, and head. However, the rates of motion of the head were not included in the analyses because their values were too small, being on the same order of magnitude as the accuracy of the DeepLabCut [34] tracking software (DLC tracking: 3.26 px and head motion mean: 3.15 px/s). We considered that hoverflies were positioned exactly at a pressure node enabling levitation. When that was not the case, hoverflies could experience passive movements due to the activation or deactivation of levitation forces. These movements are transient artifacts, vanishing as soon as the hoverflies stand in one pressure node. To avoid any possible bias in the analysis due to these artifacts, we removed 1 s of video at each change of state of the levitator (activation or deactivation). Flies were filmed in the box with the camera setup described before.

### 2.5 Statistical Analysis

The flies were tracked using Deeplabcut [34, 35]. A model was trained to recognize and track the four distinct parts of the fly's anatomy depicted in Figure 3E: the head, the abdomen, the hind legs, and the wings oriented toward the camera. The analysis of the movements subjected of the air puff was carried out over the 5 s following the activation of the air puff. All analyses were conducted using the R software (version 4.2.1). The statistical analyses were carried out on the mean rate of motion of the four body parts of each fly: this was done by calculating the Euclidean distance (in pixels) between two frames for each video footage, then we calculated the mean distance and multiplied it by the fps of the camera to obtain the linear speed in pixels per second for each fly (Figure 3F). We also analyzed three distinct parameters of the wingbeat: (i) the occurrence of the wingbeat initiation, (ii) the latency to the first wingbeat, and (iii) the total duration of the initial wingbeat sequence.

A principal component analysis (PCA) was conducted to investigate the overall behavioral differences between hoverflies when exposed to ultrasound. The validity of group discrimination was confirmed through a Wilcoxon test. Linear mixed‐effects models (LMMs) were used to investigate the influence of ultrasound on the rate of motion of each body part. Generalized linear mixed‐effects models (GLMMs) were used to analyze the occurrence of wingbeat initiation (GLMM Poisson) and the duration of the first wingbeat sequence (GLMM gamma). For each model, we removed the outliers (outliers were calculated as a residual greater than three times the standard deviation of the model's residuals). To analyze the latency before the first wingbeat onset, we performed a Cox survival analysis.

## 3 Results

As described in Section 2.5, we present our analysis of a possible effect of the ultrasound (US) emitted by the acoustic tether on the fly's behavior, which was quantified here by measuring finely the wingbeat and the movement of three natural markers along the fly's body (see Figure 3): the leg, wing, and abdomen.

### 3.1 Effect of Ultrasound Tethering on Flies' Behavior

#### 3.1.1 Analysis of Ultrasound Effect on Global Behavior

**FIGURE 4:**
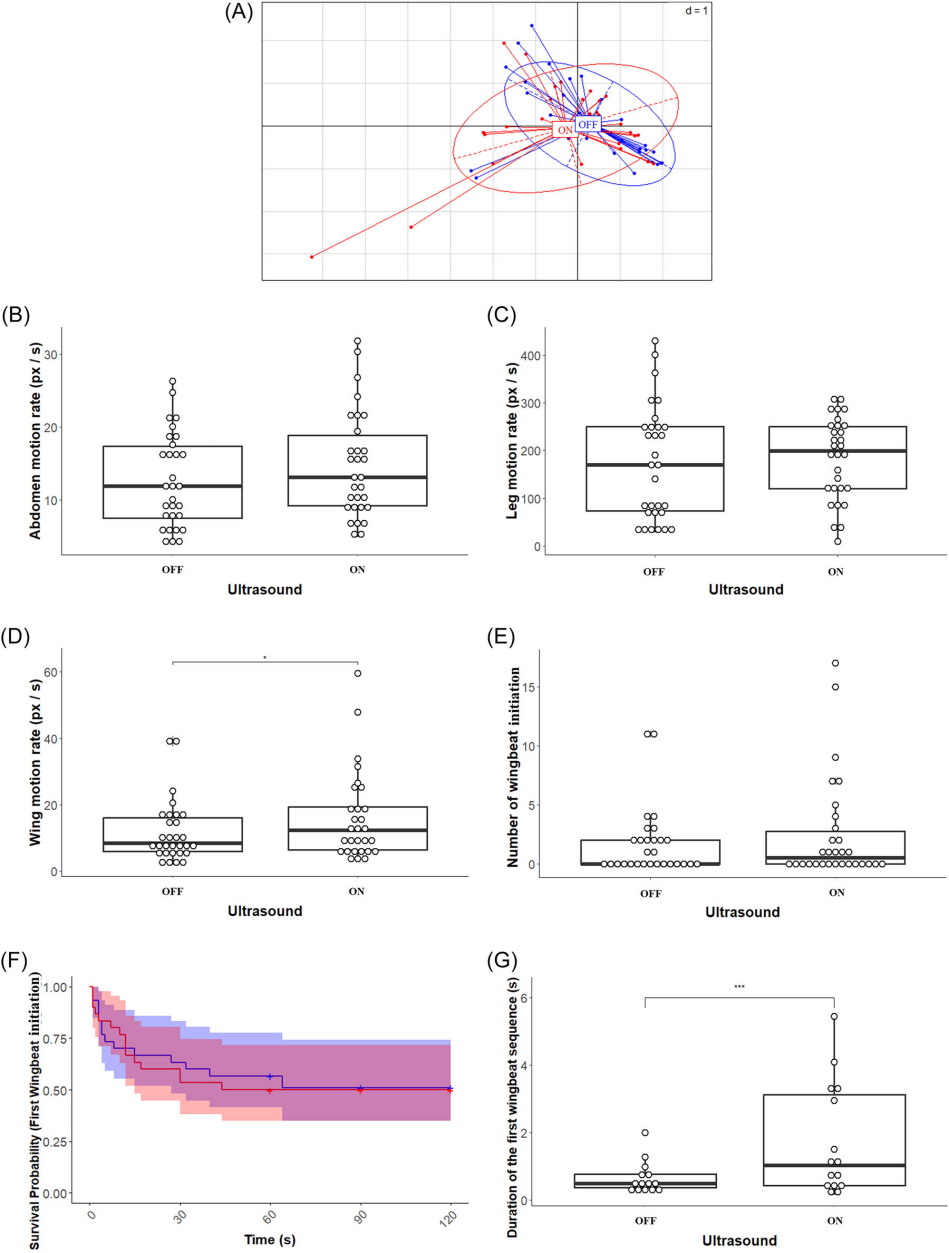
Principal component analysis of the hoverflies' four behaviors (abdomen motion rate, leg motion rate, wing motion rate, and the occurrence of wingbeat initiation). The amount of motion rate of hoverflies between two frames depended on the presence of ultrasound and wingbeat analysis. *p < 0.05; ***p < 0.001. (A) Plot of the PCA; hoverflies subjected to levitator ultrasound are in red (“ON”) and hoverflies without ultrasounds are in blue (“OFF”). (B) Amount of abdomen motion rate. (C) Amount of leg motion rate. (D) Amount of wing motion rate. (E) Occurrence of wingbeat initiation. (F) Cox survival analysis of the latency of the first wingbeat onset. Hoverflies subjected to levitator ultrasound are in red and hoverflies without ultrasound are in blue. (G) Duration of the first wingbeat sequence.

In order to compare behavior with and without ultrasound, we attached the hoverfly to a rigid tether in the middle of the acoustic levitator and filmed its movement from the side. We performed a PCA by considering the three markers and the occurrence of wingbeat initiation in order to discriminate between the two classes, with and without ultrasound (Figure 4A). The PCA projection of the data reveals intermixed classes (small interclass variance) and are statistically not different (Wilcoxon test, *W* = 530, *p*‐value = 0.242, 95% CI = [−0.315, 1.149]). Thus, PCA is unable to discriminate between the different classes (with/without ultrasound), although the first two components of PCA explain 87.9% of the variance.

#### 3.1.2 Analysis of Ultrasound Effect on Individual Behavior

Concerning the effect of ultrasound on abdomen motion rate (Figure 4B), our model shows no effect of ultrasound on this behavior and explained 34% of the variability (LMM; estimate = −1.0089±0.7248; *t* = −1.392; *p*‐value = 0.175; adj. $R^{2}=0.34$). Similar to the abdomen motion rate, the model applied to leg motion rate versus ultrasound (see Figure 4C) does not show any significant difference between the ultrasound and nonultrasound groups, and the variation observed was 62% explained by the model (LMM; estimate = −6.003±8.265; *t* = −0.726; *p*‐value = 0.4676; adj. $R^{2}=0.6219$). To complete the analysis of the different markers one by one, we studied the motion rate of the wings between two consecutive frames (Figure 4D). The linear mixed‐effect model explained 50% of the variation observed and revealed a slight effect of ultrasound (LMM; estimate = −2.348±1.088; *t* = −2.159; *p*‐value = 0.0393; adj. $R^{2}=0.5005$). Flies subjected to ultrasound moved their wings significantly more than flies not subjected to ultrasound.

For the wingbeat analysis, we started by counting the occurrence of wingbeat initiation (Figure 4E). Our model did not show any significant difference between the ultrasound and nonultrasound groups, and explains 94% of the variation (GLMM Poisson; estimate = −0.08966±0.09575; *z* = −0.936; *p*‐value = 0.349; adj. $R^{2}=0.9424$). We then studied the latency of the first wingbeat onset (Figure 4F). The Cox survival analysis does not show any significant difference (mixed‐effects Cox model; coef = 0.07145±0.42748; *z* = 0.17; *p*‐value = 0.867). The latency to the first wingbeat onset is the same for flies exposed to ultrasound and flies not exposed to ultrasound. Furthermore, for both groups, only 50% of the flies triggered the wingbeat during the experiments and half of the wingbeat triggered within the first 12 s following the activation or deactivation of the ultrasound. Finally, we studied the duration of the first wingbeat sequence (Figure 4G). The GLMM showed a significant difference between the two groups (GLMM gamma; estimate = 0.35562±0.08183; *t* = 4.346; *p*‐value < 0.001; adj. $R^{2}=0.6964$): the first wingbeat sequence is longer in flies exposed to ultrasound.

#### 3.1.3 Analysis of Air Puff Coupled With Ultrasound

With tethered insects, air puffs are usually used to trigger the wingbeat and even help to sustain prolonged flight. However, unlike *Drosophila* or the locust, the hoverfly here did not systematically trigger its wingbeat in response to an air puff directed toward its head: only 6 out of 30 flies triggered a wing beat reflex. Despite the low occurrence of this reflex in tethered hoverflies, we performed the same analysis for data relating to wingbeat (see Supporting Information). We found no significant differences between the groups exposed to ultrasound and those that were not.

### 3.2 Acoustic Tethering of Other Insect Species

In this study, we have demonstrated that the behavior of hoverflies is not affected by the acoustic pressure inside the levitator. We then wondered whether it would be possible to use the latter with different insect species. To answer this question, we tested the levitator with several insects of various sizes. We focused on diurnal insects because we know that nocturnal insects like moths (Noctuoidea) are sensitive to ultrasound emitted by predators like bats [21]. The typical escape behavior in moths consists in cessation of flight and dropping to the ground, and thus the levitator cannot be used for these insects.

**FIGURE 5:**
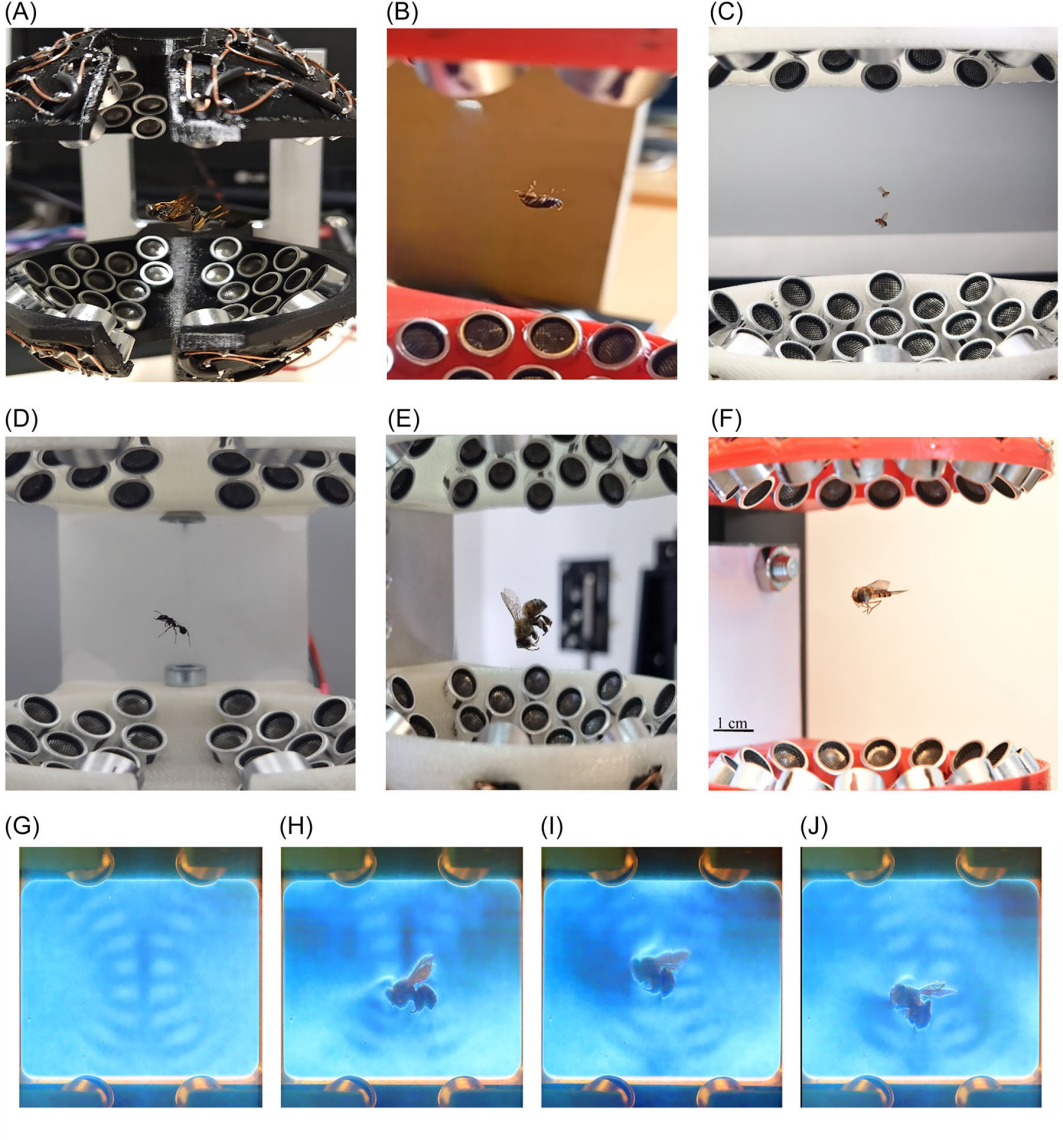
Photographs of different insects levitating. (A) Dead mud‐dauber wasp (49 mg) levitating upside‐down. (B) Dead *Blattella germanica* levitating (4 mg). (C) Two dead *Drosophila melanogaster* levitating (<1 mg). (D) Live ant levitating (7 mg). (E) Dead bee levitating (58.6 mg). (F) Hoverfly levitating (∼30 mg). (G) Schlieren photographs of the pressure distribution of the standing wave taken as a reference for the following images. The standing wave is composed of pairs of high pressure zones (antinodes) visualized as bright spots distributed along the vertical axis. (H) An insect trapped horizontally lying on two pressure antinodes. (I) The insect is trapped in a diagonal having half of its body trapped at the vertical nodal plane and the head at an upper node‐antinode region. (J) Similar to the previous image, but the insect trapped in a different vertical position with slightly different orientation.

As shown in Figure 5, we levitated several insects of various sizes (alive and dead). Unlike the TinyLev [14], our device can levitate a wider variety of insects, like two dead fruitflies (*Drosophila melanogaster*; <1 mg) in two different pressure nodes (Figure 5C; Videos S6 and S7). The largest insects levitated here were a dead bee (*Apis mellifera*) weighing 58.6 mg (Figure 5E; Videos S8–S10) and an upside‐down dead mud‐dauber wasp (*Sphecidae sp*.) weighing 49 mg (Figure 5A). The technique was also highly effective in levitating various living insects in a stable position (right side up or upside‐down) without them flying away or struggling erratically: an upside‐down German cockroach (*Blattella germanica*; 10 mg) (Figure 5B), an ant (*Formicidae* sp.; 7 mg) (Figure 5D; Videos S11–S14) and the hoverflies used in the experiment (∼30 mg) (Figure 5F).

## 4 Discussion

We have shown here that acoustic levitation can be useful for levitating insects of various sizes (ranging from 1 to 25 mm [∼3λ]) and various masses (ranging from <1 to 58.6 mg). In future experiments, acoustic levitation will be employed to prevent flies from relying on leg proprioception prior to free fall, thereby enabling an unbiased assessment of their orientation relative to gravity. Unlike earlier studies conducted in the laboratory, where flies either maintained contact with the ceiling of the chamber [4, 36] or were suspended with their legs dangling [3], acoustic tethering allows manipulation of the fly's initial orientation (right‐side up or upside‐down) before the fall without stimulating leg proprioception.

### 4.1 Behavioral Impact

The time for the levitator to reach full power and for the acoustic pressure field to stabilize is about 2 ms, which probably makes the fly unable to sense any difference during this fast transition period occurring during the activation (or deactivation) of the US, only if the hoverflies are perfectly positioned in the pressure nodes (which allow levitation). Our behavioral tests of the levitation technique showed that the levitator had little effect on the behavior of the hoverflies: only the wing motion rate and the first wingbeat duration are higher for flies subjected to US. The slight increase in wing movement amplitude and the increase of the wingbeat duration might indicate a perception of the US and an attempt to escape. However, the occurrence of wingbeat initiation is not significantly different with or without ultrasound (Figure 4E). In addition, this increase could be also due to an unstable position of the hoverfly within the pressure nodes, because of passive motion, which could make the fly increase its wing movement so as to be stabilized between the pressure antinodes. As our analyzes did not reveal any other behavioral differences (overall behavior, individual behavior, and air puff), the only difference might therefore due to the fact that hoverflies were not exactly positioned between the pressure nodes when the ultrasound was activated/deactivated. This unstable position could lead to greater wing movements, which is not the case when the insect levitates (unpublished observations).

Finally, there remains a classic behavior in stressful conditions that we cannot measure directly, which is named “freezing” [37]. The fact that hoverflies can move at all times during ultrasound emission, and that the most of the tests did not reveal any differences between the absence/presence of US, allows us to confirm that the absence of movement is not due to stress caused by the test. Moreover, given that the behaviors during US is the same as in the periods without US (except wing movements and wingbeat duration), one can assume that US does not induce freezing behavior and thus no ceiling effect.

### 4.2 Perspectives on Levitation

Our levitator was designed to levitate various insects, dead or alive, right‐side up or upside‐down, of different sizes and shapes, with high spatial stability (i.e. without oscillating, vibrating, or rotating) for a long time. Flying insects frequently initiate flight from various orientations, such as taking off from the underside of foliage. Our experimental setup seeks to replicate these ecologically relevant conditions in a laboratory setting, allowing for a more robust analysis of the fly's aerial righting reflexes and upside‐down takeoff maneuvers. However, some insects were able to fly away if they were strong enough, which reduced the levitation time. This ability of some insects to escape by flying means that they have enough strength to overcome acoustic forces, and must be calm for successful trapping.

The schlieren technique allows to clearly visualize the pressure distribution of the standing waves in our device (Figures 3C and 5G), which has cross‐shaped nodal regions around the center of the levitator. By levitating a single dead bee, we observed that it can be trapped at different positions and orientations along these cross‐shaped nodal regions. In some cases (Figure 5H), the insect is trapped with the body in a horizontal position along two antinodes. In other cases (Figure 5I,J), half the insect body is trapped at the axial nodal plane, while the head seems to be trapped at an antinode. According to our experiments, the levitation behavior was found to differ according to the length and shape of the object: large objects (diameter > 0.5 λ, i.e., 4.3 mm) levitate at the antinodes between several pressure nodes and shift the object with respect to the vertical axis of symmetry [11].

Our device was able to levitate an insect weighing 58.6 mg. However, heavier nonspherical objects can be levitated, such as electronic resistors weighing 149.8 mg [15]. Levitation of large objects may shift the resonant condition, so, stable levitation of large and heavy insects may require a distance adjustment between the domes using the schlieren technique [31]. By optimizing levitation, heavier living insects such as bees, houseflies, and small beetles could be kept in levitation. On the other hand, insects such as ladybugs could be kept alive in levitation without being able to fly away, as has been shown with a single‐axis resonant acoustic levitator [12].

Attachment of tissue using beeswax and rosin requires direct cuticular manipulation, potentially reducing the benefits of a contactless tether. The possibility to put insects in levitation without attachment is completely feasible. To achieve this, the insect must be anesthetized with CO2 or cold and placed in levitation with an entomological clamp. The anesthesia will allow the insect to be positioned without it clinging to the clamp, and the levitation is stable enough to allow the insect to be held until it is fully awake.

Further experiments could thus be conducted without the constraints imposed by a tether. Acoustic tethering opens new avenues for investigating the behavior of insects of different shapes, sizes, and masses, as it enables precise control over the trapping force and rotational dynamics of the levitating animal. Moreover, following extended assessments of the levitator's effects, it is conceivable that this device could be integrated into a flight simulator [38] to study the optomotor response of *Drosophila* under conditions free from friction and additional inertia associated with tethering.

## 5 Conclusion

Here, we have demonstrated that acoustic levitation is effective for levitating insects across a wide range of body sizes (1–13.5 mm) and masses (<1– 58.6 mg). Importantly, levitation does not appear to affect hoverfly behavior; the minimal impact observed may stem from misalignments in pressure nodes caused by the use of a mechanical tether here, an artifact absent under free levitation. In further experiments, acoustic levitation will be used to eliminate the influence of leg proprioception prior to free fall. These initial trials will then be compared against control data available in the literature [3, 36], enabling us to model graviception in flight. Overall, acoustic tethering opens promising new avenues for behavioral studies in insects.

## Author Contributions

T.G. and S.V. drew up the research project. T.G. performed the experiments. T.G., D.M., and S.V. analyzed the data. V.C. designed and made the levitator. T.G., V.C., D.M., and S.V. wrote the paper.

## Disclosure

The authors have nothing to report.

## Conflicts of Interest

The authors declare no conflicts of interest.

## Supporting information




**Supporting Material**: nyas70281‐sup‐0001‐SuppMat.pdf
