# Supplementary material for: Contactless Acoustic Trapping of Hoverflies for Behavioral Studies
Source: Ann N Y Acad Sci. 2026 May 18;1559:e70281. doi: 10.1111/nyas.70281 (PMC13182685; doi:10.1111/nyas.70281)
Supplement: Supplementary file 1 — Supporting Material: nyas70281‐sup‐0001‐SuppMat.pdf [file NYAS-1559-0-s001.pdf]

Supplementary Information - Contactless acoustic trapping of hoverflies for behavioral studies

Gaillard Thomas[1], Contreras Victor[2], Martinez Dominique[1], Viollet Stéphane[1]

[1]Aix Marseille University, CNRS, ISM, Marseille, France

[2]Universidad Nacional Autonoma de Mexico, Instituto de Ciencias Fisicas, Cuernavaca, Mexico

Data and videos used in this article are available on-line at: https://osf.io/748sb/overview?view_only=cda82776f5ba44988a28ea45deabde2d

1 Results

1.1 No effect of ultrasound tethering on flies' behavior

1.1.1 Analysis of air puff coupled with ultrasound

With tethered insects, air puffs are usually used to trigger the wingbeat and even help to sustain prolonged flights. However, unlike *Drosophila* or locust, hoverfly here did not trigger systematically its wingbeat in response to an air puff sent toward its head: only 6 out of 30 flies triggered a reflex wing beat. Despite the low occurrence of this reflex in tethered hoverflies, we performed analysis for wingbeat data with air puffs.

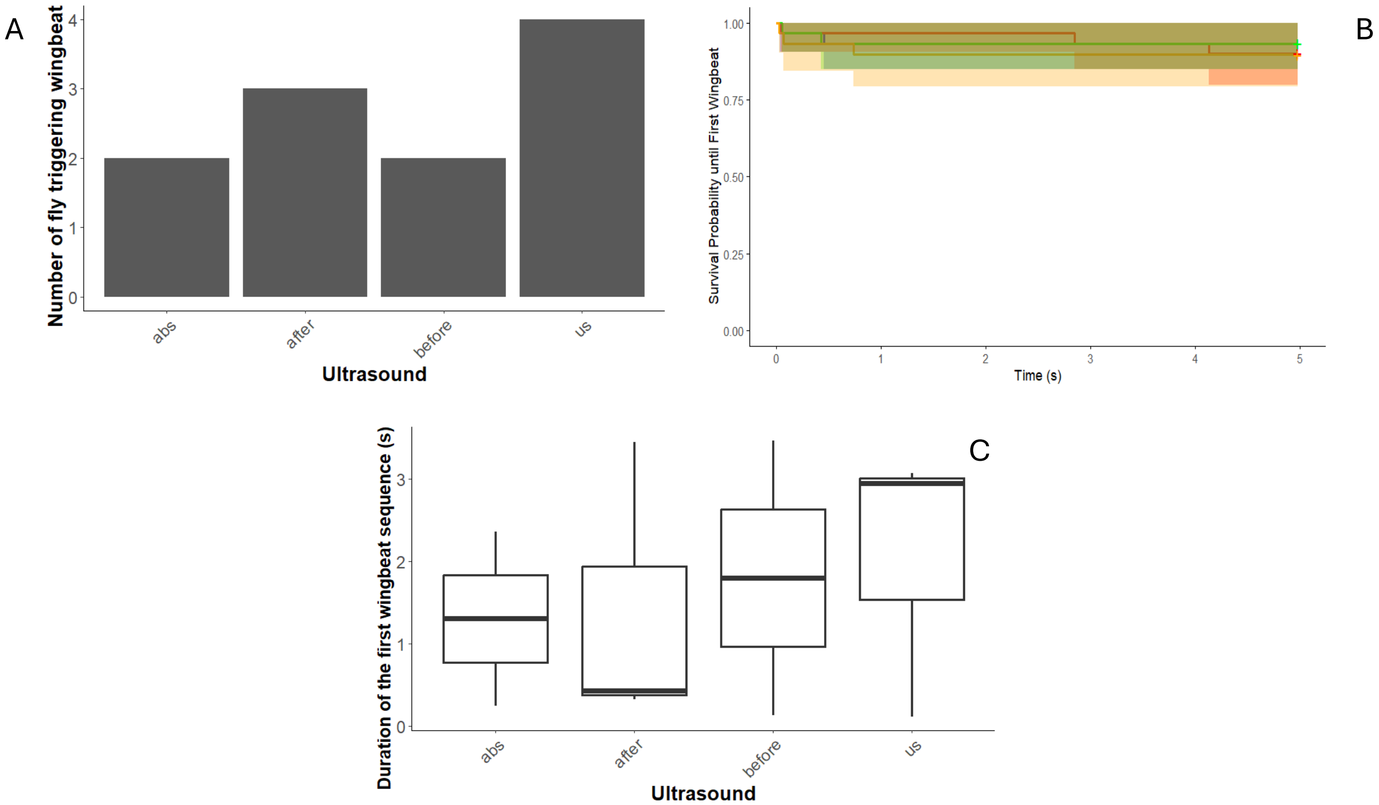

Supplementary Figure S1: Results of ultrasound effect mixing the air puff. (A) Barplot the occurrence of wingbeat initiation after airpuff. (B) Cox survival analysis of the latency of the first wingbeat initiation after airpuff. Airpuff with ultrasound are in orange, airpuff without ultrasounds are in blue, airpuff before the experiments are in green and airpuff after the experiments are in red. (C) Duration of the first wingbeat sequence after airpuff.

Concerning the wingbeat analysis mixed with air puff, we count the occurrence of wingbeat initiation (Fig. S1 A). Our model does not show any significant difference (GLMM Poisson; p-value = 0.888) between the groups (before the experiments, after the experiments, with ultrasound and without ultrasound).

We then studied the latency of the first wingbeat initiation (see Fig.S1 B). The cox survival analysis does not show significant difference (Mixed effects cox model; p-value > 0.05). The latency to the first wingbeat initiation is the same for flies before, after experiments, exposed to ultrasound and flies not exposed to ultrasound.

At last, the GLMM for the duration of the first wingbeat sequence (see Fig.S1 C) shows no significant difference between the four groups (GLMM Gamma; p-value = 0.58861).
